# Brain infarction following meningioma surgery—incidence, risk factors, and impact on function, seizure risk, and patient-reported quality of life

**DOI:** 10.1007/s10143-022-01840-1

**Published:** 2022-07-28

**Authors:** Per S. Strand, Lisa M. Sagberg, Sasha Gulati, Ole Solheim

**Affiliations:** 1 Department of Neuromedicine and Movement Science, Norwegian University of Science and Technology, Trondheim, Norway; 2 Department of Neurosurgery, St. Olavs Hospital, Trondheim University Hospital, Trondheim, Norway; 3 Department of Public Health and Nursing, Faculty of Medicine and Health Sciences, Norwegian University of Science and Technology, Trondheim, Norway

**Keywords:** Meningioma, Outcome, Infarction

## Abstract

In this study, we seek to explore the incidence of and potential risk factors for postoperative infarctions after meningioma surgery, in addition to the possible association with new neurological deficits, seizures, and health-related quality of life (HRQoL). A single-center cohort study was conducted, where all patients operated for an intracranial meningioma at our institution between 2007 and 2020 were screened for inclusion. Clinical data were prospectively collected in a local tumor registry, and HRQoL was assessed using both generic and disease-specific instruments. In total, 327 meningioma operations were included, and early postoperative MRIs showed peritumoral infarctions in 114 (34.9%). Median infarction volume was 4.5 ml (interquartile range 2.0–9.5) and 43 (37.7%) of the infarctions were rim-shaped, 44 (38.6%) were sector-shaped, 25 (21.9%) were a combination of rim- and sector-shaped, and two (1.8%) were remote infarctions. Permanent neurological deficits were seen in 22 patients (6.7%) and deficits were associated with infarctions (*p* < 0.001). There was no difference in frequency of registered postoperative epilepsy between patients with versus without infarctions. Patients with infarctions reported more future uncertainty; otherwise, there were no significant differences in disease specific HRQoL between patients with versus without infarctions. In this study, we found that peritumoral infarctions after meningioma resection are common. Most patients with permanent neurological deficits had infarctions. Yet, most infarctions were small, and although sometimes symptomatic on individual level, infarctions did not lead to significant deterioration of HRQoL on group level.

## Background

Meningioma is the most common primary intracranial tumor [6, 22], with a reported prevalence of 0.9% and 1.0% in population-based MRI studies [11, 33]. Patients with growing or symptomatic tumors are often offered surgery. Although patients operated for meningioma exhibit good tumor control and survival rates [17, 31, 35], potential benefits of surgery and the intraoperative choices must be balanced against the risk of new or worsened neurological deficits and postoperative complications.

Postoperative complications after meningioma surgery may be associated with decreased survival [14], and 4–14% experience neurological deterioration following surgery [2, 14, 24]. The risk of surgically induced epilepsy is 13–19% [15, 34], and emotional and cognitive difficulties following meningioma surgery have been reported in up to 40%, as well as frequent problems with postoperative fatigue [19, 32]. In Sweden, the proportion of patients working 2 years after meningioma surgery was only 57% compared to 84% in matched controls [29].

Although there is an increased awareness of the importance of peritumoral infarctions related to glioma surgery [1, 4, 8, 12, 23, 26, 28], peritumoral infarctions after meningioma resection are less studied and the main focus has been on venous infarctions [27]. However, a recent publication with 122 patients found that an area with MR restricted diffusion over 1 cm was seen after 22% of meningioma operations and was associated with postoperative deficits [16]. A recent study found that postoperative ischemia, defined as ischemia/infarction found on postoperative MRI not related to normal postoperative changes within the resection bed, occurred in 13 of 57 (23%) cases and was associated with postoperative seizures [7].

In this single-center prospective cohort study, we sought to study the incidence of and potential risk factors for postoperative infarctions after meningioma surgery as well as the possible association with new neurological deficits, seizures, and health-related quality of life (HRQoL).

## Methods

### Study cohort

We screened all adult patients (≥ 18 years) operated for intracranial meningioma at the Department of Neurosurgery at St. Olavs Hospital, Trondheim, Norway, from January 2007 through May 2020 with available postoperative MRI including DWI sequences taken less than 72 h after surgery. Patients with a postoperative CT-control only, with a postoperative MRI control done longer than 72 h after surgery, or without a histologically confirmed diagnosis were excluded.

### Data collection

Patient characteristics were collected prospectively in a local tumor registry. Tumor grading was done by a neuropathologist. Meningiomas operated before the last half of 2016 were classified according to the WHO 2007 classification, and the WHO 2016 classification was used thereafter. Preoperative tumor volumes were segmented semi-automatically from pre-treatment MRIs. Surgical extent of resection was classified from postoperative MRI scans into gross-total resection (radiologically total tumor resection on early postoperative MRI) or subtotal resection (tumor remnants seen on early postoperative MRI). Starting in 2015, operating surgeons filled out a questionnaire immediately after surgery where they reported on intraoperative tumor characteristics. Neurological deficits were defined as either a permanent paresis or permanent dysphasia/aphasia registered at follow-ups 4 weeks or longer after surgery and were retrospectively retrieved from electronical medical records.

**Fig. 1:**
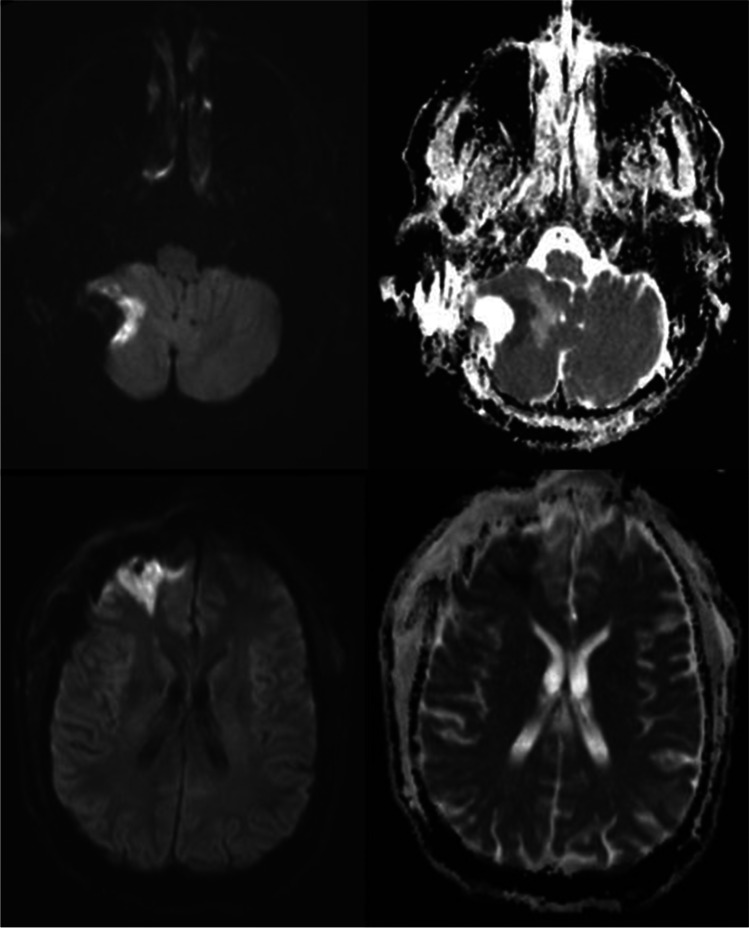
Top row shows B1000 and ADC series, from left to right, respectively, of a rim-shaped infarction, whereas bottom row displays a sector-shaped infarction

Our postoperative MRI protocols and routines for segmentation of infarctions and tumors have been described in detail earlier [26]. Briefly, infarctions were detected using postoperative B100 sequences on diffusion-weighted images (DWI) and apparent diffusion coefficient (ADC) maps. Areas with high signals on B1000 images and corresponding low values on ADC maps, which could not be explained by other diffusion abnormalities, and with a thickness of at least 3 mm were defined as infarctions. Infarctions were classified as either rim-shaped, sector-shaped, remote, or a combination of sector- and rim-shaped. Examples of sector-shaped and rim-shaped infarctions are presented in Fig. 1. All infarctions were manually segmented using DWI, whereas tumors were segmented semi-automatically using preoperative T1-weighted images with contrast.

Following 112 of the operations from 2015 and onwards, surgeons reported on a questionnaire immediately after surgery whether or not there had been a certain or probable intraoperative injury to a functional artery during surgery.

### Quality of life

One to 3 days before surgery, the patients completed HRQOL questionnaires on. In cases where the patient was unable to complete the questionnaire (for instance because of cognitive impairment or language deficits), a study nurse or a next of kin helped. Such proxy ratings in neuro-oncological patients have shown good agreement with the patients’ own scores [9, 25]. A 1-month follow-up of HRQoL was done as a structured telephone interview conducted by a study nurse.

EQ-5D 3L is a generic questionnaire designed by the EuroQol Group and consists of a descriptive system and a visual analog scale (VAS) [5]. The descriptive system covers five dimensions, mobility, self-care, activity, pain, and anxiety, scored as either “no problem,” “slight problem,” or “major problem.” EQ-5D 3L is used for numerous conditions and has been validated for the Norwegian population [13, 20]. One patient had four missing domains in the preoperative questionnaire and was excluded. Six patients had one missing item on preoperative questionnaires, five of these were imputed as “no problem” as they reported “no problem” in all but one missing domain, whereas one responder reported “slight problem” in all but one missing domain and in this case “slight problem” was imputed. Two patients had one missing item on postoperative questionnaires, and both were imputed as “no problem” as they reported “no problem” in all other domains.

EORTC QLQ-C30 is a frequently used disease-specific questionnaire to score HRQoL in cancer patients and is used to assess symptoms and functional outcomes for many cancers [10, 37]. It covers central domains in daily life, for instance physical, emotional, and role functioning, in addition to symptoms common to all cancers, such as pain, nausea, fatigue, and loss of appetite.

EORTC BN20 is a supplement for patients with brain cancer, to be used in combination with QLQ-30 [21]. It compromises four multi-item scales of future uncertainty, visual impairment, motor dysfunction, and communication problems. In addition, there are seven single-item scales on symptoms related to brain cancers. Data from EORTC QLQ-C30 and BN20 were only available from 2015 and onwards, and no imputations for missing data were done for these disease-specific instruments.

### Statistical analyses

Statistical analyses and descriptive statistics were performed with IBS SPSS Statistics version 27.0 (IBM, Armonk, NY). Q-Q plots were used to assess normal distribution. A binominal logistic regression model was constructed to establish risk factors for infarctions, and for these analyses included variables were chosen based on presumed relevance. Fisher’s exact tests were applied for calculations of neurological deficits due to a small number of events. Statistical significance level was set to *p* ≤ 0.05.

### Ethics and approval

All patients provided written informed consent to be included in research, and the study was approved by the regional ethics committee (REK) (reference 2018/1187). Data collection was done according to the guidelines of the Helsinki Declaration.

## Results

We identified 422 patients with meningiomas who underwent 467 operations, of which 327 (70.2%) had an early postoperative MRI, 118 (25.3%) only had a postoperative CT scan, and 22 (4.7%) underwent no early postoperative imaging control.

Among the 327 meningioma operations with early MRI scans, 114 (34.9%) showed peritumoral infarctions. Median total infarction volume was 4.5 ml (interquartile range 2.0–9.5). Forty-three (37.7%) of the infarctions were rim-shaped, 44 (38.6%) were sector-shaped, 25 (21.9%) were a combination of rim- and sector-shaped, and two (1.8%) were remote infarctions. Median infarction volumes in different subgroups were 2.3 cm^3^ (interquartile range [IQR] 1.3–3.1), 8.3 cm^3^ (IQR 4.3–13.2), 8.2 cm^3^ (IQR 3.5–10.7), and 0.27 cm^3^ for rim, sector, combined sector and rim, and remote infarctions, respectively. Postoperative infarctions were more common is surgeons reported intraoperative damage to a functional artery, 17 of 38 (44.7%) vs 12 of 74 (16.2%), *p* = 0.003.

A binominal logistic regression that predicts the likelihood of postoperative infarctions is presented in Table 1. As seen, increasing age is associated with an increased likelihood of postoperative infarctions, and there was a non-significant trend that larger tumors were associated with postoperative infarctions.

**Table 1:** Risk factors for postoperative infarctions

Covariate	OR	95% CI	*p* value
Male sex	1.22	0.72–2.05	0.460
Age	1.03	1.01–1.05	0.001
Tumor histology			0.869
WHO grade 1	1.18	0.98–14.28	0.896
WHO grade 2	1.37	0.11–16.96	0.807
WHO grade 3	Reference		
Tumor location			0.255
Convexity	0.411	0.17–1.03	0.570
Parasagittal or falcine	0.419	0.17–1.06	0.660
Supratentorial skull base	0.514	0.22–1.23	0.135
Infratentorial	Reference		
Preoperative tumor volume (cm^3)^	1.007	0.99–1.02	0.083
Primary operation	0.735	0.36–1.49	0.393
Gross total tumor resection	0.766	0.42–1.41	0.389
ASA			0.488
1	1.322	0.30–5.83	0.713
2	1.438	0.79–2.61	0.232
3	Reference		
KPS ≥ 70	0.815	0.33–2.04	0.663

### New neurological deficits

Acquired language or motor deficits are presented in Table 2. As seen, neurological sequelae were seen in 22 patients (6.7%) and were more common in patients with infarctions. Among 6 patients without infarctions who still experienced permanent deficits after surgery, one patient had a postoperative hematoma that required urgent surgery, and five remaining patients had parasagittal meningiomas affecting the primary motor cortex and likely had direct damage to the underlying primary motor cortex during dissection. There was no difference in frequency of registered postoperative epilepsy between patients with versus without infarctions, 5.1% vs 3.6 (*p* = 0.549), respectively.

**Table 2:** New neurological deficits in patients with versus without infarctions

	Infarction *N* = 99	No infarction *N* = 192	Fisher’s exact test
Permanent deficits	16 (72.7%)	6 (27.3%)	*p* < 0.001
No or only transient deficits	84 (31.2%)	185 (68.8%)	

### Quality of life

**Fig. 2:**
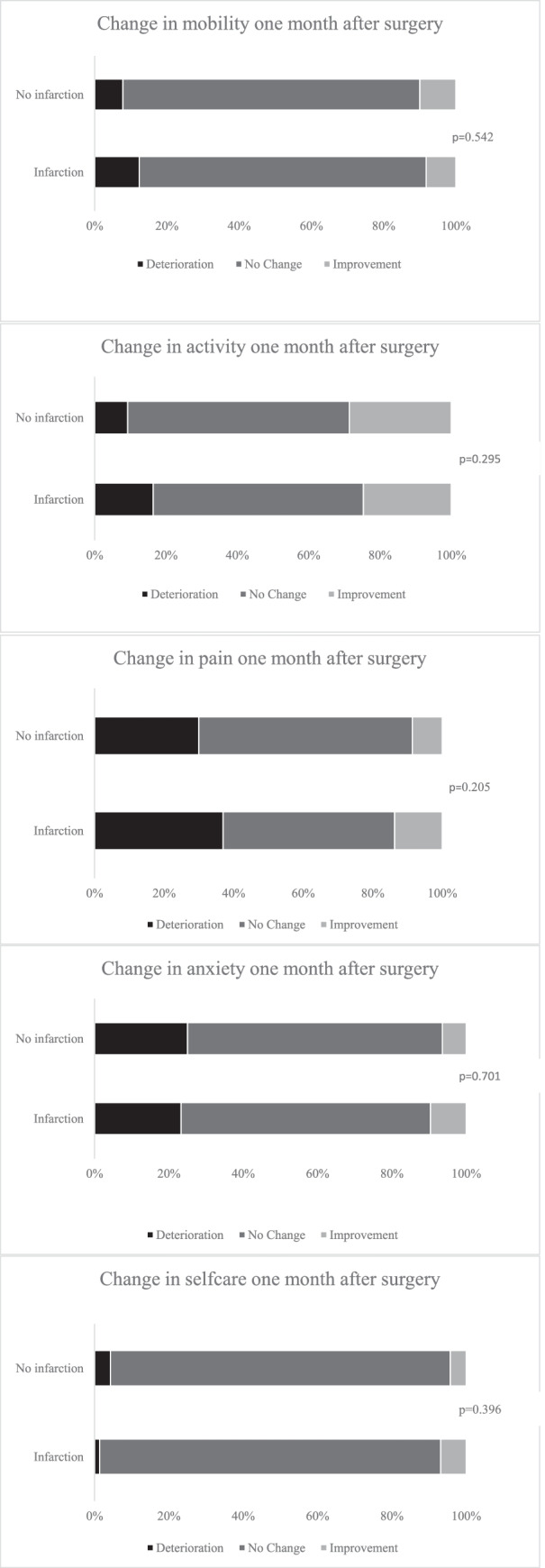
Changes in EQ5D domains 1 month after surgery

In total, 227 and 225 completed the EQ-5D questionnaires before and after surgery, respectively. Patients reported an overall better quality of life after surgery, with a median index value at 0.796 before surgery (IQR 0.689–0.848) and 0.812 one month after surgery (IQR 0.714–1.00), *p* = 0.001. There was no significant difference in change in EQ5D-index value 1 month after surgery in patients with versus without infarctions (median change 0.052 (IQR − 0.071–0.20) vs 0.0 (IQR − 0.36–0.16), *p* = 0.435). In Fig. 2, the distributions of change for each dimension in EQ5D from before and to 1 month after surgery are presented. As seen, there were no significant differences between patients with versus without infarctions in the five EQ5D domains, and most patients reported no change in mobility, self-care, pain, anxiety, or activity 1 month after surgery.

For the EORTC QLQ-C30 and EORTC QLQ-BN20 instruments, 141 patients completed the questionnaires. Patients with infarctions reported more future uncertainty (median change from baseline 16.7 (IQR 8.3–41.6) vs 8.3 (0–41.6), *p* = 0.048). Otherwise, there was no statistical difference in the functional or symptom scales when comparing patients with and without infarctions.

## Discussion

In this single-center population-based cohort study, we found that brain infarctions are common after meningioma surgery and are associated with higher risk of permanent neurological deficits. However, most infarctions are functionally silent and there is no significant effect on patient-reported HRQoL on group level. Patients with postoperative infarctions were older than patients without infarctions.

The risk of infarctions following meningioma surgery is high, and mean infarction and mean infarction volumes are even larger than for glioma operations [22]. The vascular supply of the aging brain is more vulnerable and, like in glioma surgery, the risk of infarctions is higher with age [22]. Many meningiomas involve underlying cortical vessels, and may even engulf larger arteries, and damage to both arteries and veins may occur inadvertently and may sometimes be unavoidable during the dissection. Also, sometimes vascular supply of meningiomas may in part be shared by the cortex and the tumor, adding to the risk of resection. In larger tumors, more vessels are often at risk and visual control of vessels may be poorer if the tumor is resected en bloc. Larger meningiomas tend to recruit more blood supply from the underlying brain, and visual control of underlying normal vessels can be difficult even after extensive debulking, especially in firmer tumors. Thus, as perhaps expected, we found a trend that risk of infarctions was larger in larger tumors. However, in contrast to our findings, a recent study found that tumor size was not associated with significant infarctions, defined as DWI signal larger than 1 cm [16]. This might be explained by a smaller sample size and that only convexity meningiomas were included in that study. Nevertheless, as pointed out by the authors, how adherent a meningioma is to cerebral cortex might be more challenging than the size of the meningioma itself. Still, we did not find a significant lower risk of infarctions in WHO grade 1 meningiomas that per definition do not infiltrate normal tissue and more seldom exhibit peritumoral edema.

The risk of infarctions is naturally higher, but not 100%, if the surgeon reported sacrifice of an assumed normal artery, reflecting that collateral blood supply can be unpredictable. Surgeons should strive to develop dissection techniques that limit the risk of infarctions. In the present study, we did not assess the potential effect of different surgical techniques and tools, yet these factors may impact the likelihood of postoperative infarctions. Increasing awareness among neurosurgeons and neuroradiologists of the importance of DWI changes on early postoperative MRIs and inclusion of these sequences in routine assessments may in itself be a step forward towards developing better and safer surgery, and individual surgeons should attempt to learn from their postoperative MRI findings to make potential adjustments in their technique. In vulnerable regions, or in tumors that engulf important vessels or are very adherent, subtotal resections and/or stereotactic radiosurgery may be a viable alternative.

Most patients operated for meningioma have good survival rates and most meningiomas are slow-growing tumors. Although the potential for rehabilitation is larger with increased survival, weighing potential benefits of tumor resection against risk of surgically induced permanent deficits is important in preoperative planning, and the risk of peritumoral infarctions should add to the preoperative evaluation of surgery-related risks. Although both definitions of deficits and time of assessment vary across studies, the risk of neurological deficits following meningioma surgery is significant. In a Swedish national registry-based study, 14.8% developed new neurologic deficits following meningioma surgery while 8.3% of preoperative asymptomatic patients developed deficits [3]. A large Norwegian population-based cohort study found that new postoperative neurological deficits occur in only 4% of meningioma resections, and that postoperative hematoma was the only significant predictor for postoperative neurological deterioration [14]. Although postoperative hematomas can lead to new sequelae, the present study indicates that peritumoral infarctions may be an important and perhaps less acknowledged cause of postoperative deficits. As seen, the majority of patients with acquired postoperative deficits have surgery-related infarctions.

Still, some patients acquired neurological deficits without infarctions or hematomas, and the second most common cause of new deficits may be direct cortical damage during dissection. However, the sensitivity of DWI sequences for detecting very small infarctions may be low as the slice thickness is often several millimeters, and artifacts, for example, from clamps and plates used for bone flap fixation or small hematomas, may hamper interpretation. In the present study, we used a 3-mm cut for significant infarctions, as done in earlier studies [12, 26], yet smaller infarctions may also cause deficits if located in eloquent areas.

Unlike a recent study [7], we did not find an association between infarctions and risk of postoperative epilepsy. However, we did not account for preoperative seizures in the analyses and with longer follow-ups frequencies of seizures would be expected to increase. In a Swedish registry-based study, nearly one in five meningioma patients were still on antiepileptic drugs 2 years after surgery [30]. Thus, due to the frequent use of antiepileptic drugs postoperatively in patients with preoperative seizures, and due to the short follow-up in many studies, underestimation of postoperative seizure frequencies is likely.

In the present study, no difference in HRQoL was found between patients with versus without infarctions. HRQoL in patients harboring meningiomas is not much studied, but a systematic review found that patients reported worse HRQoL than healthy controls both prior to and after surgery [36]. The observed lack of a difference in complete case analysis of HRQoL in the two groups in our study might be influenced by the fact that data on HRQoL may not be missing at random, and may also reflect that the questionnaires used do not necessarily cover all important aspects of quality of life in patients with meningiomas [18]. Moreover, patients with larger tumors more often had infarctions, and larger tumors may have a greater preoperative impact on HRQoL, thus masquerading the true effects of peritumoral infarctions.

This study has some other limitations. As patients operated for meningiomas have good survival rates, prospective long-time data on neurological sequelae because of peritumoral infarctions would be of interest, as even permanent deficits may get better after rehabilitation. In addition, EORTC QLQ-C30 and BN20 were not collected in the entire study period, and a larger sample size with completed questionnaires might have yielded different results. Further, revisions of the WHO classification system during the inclusion period may have affected the number of patients with atypical meningiomas in the study. Strengths in this study include patient-reported measurements of quality of life, the relatively large sample size, manual segmentations of all tumors and infarctions for precise volumetric assessment, and prospective collection of clinical data.

## Conclusion

In this prospective single-center cohort study, we found that one third of the patients operated for an intracranial meningioma had infarctions detected by early postoperative MRIs. Infarctions were more common in older patients, and most patients with permanent neurological deficits had infarctions. However, most infarctions were rather small and on group level, infarctions did not lead to significant deterioration of HRQoL.

## Data Availability

Not applicable.
